# Tumor Microenvironment Responsive TPZ-Loaded Core-Shell Polymeric Nanoparticles for Selective Cancer Bioreductive Therapy

**DOI:** 10.34172/apb.025.43945

**Published:** 2025-06-16

**Authors:** Sajjad Alimohammadvand, Mohammad Shahpouri, Mohammad Amin Adili Aghdam, Hasan Majdi, Hamed Hamishehkar, Masoumeh Kaveh Zenjanab, Abolfazl Barzegari, Mehdi Jaymand, Zohreh Amoozgar, Rana Jahanban Esfahlan

**Affiliations:** ^1^Department of Medical Biotechnology, Faculty of Advanced Medical Sciences, Tabriz University of Medical Sciences, Tabriz, Iran; ^2^Drug Applied Research Center, Tabriz University of Medical Sciences, Tabriz 5166614733, Iran; ^3^Research Center of New Material and Green Chemistry, Khazar University, 41 Mehseti Street, AZ1096, Baku, Azerbaijan; ^4^Nano Drug Delivery Research Center, Health Technology Institute, Kermanshah University of Medical Sciences, Kermanshah, Iran; ^5^Student Research Committee, Kermanshah University of Medical Sciences, Kermanshah, Iran; ^6^Department of Radiation Oncology, Massachusetts General Hospital & Harvard Medical School, Boston, USA; ^7^Immunology Research Center, Tabriz University of Medical Sciences, Tabriz, Iran

**Keywords:** Hypoxia-responsive, Nanoparticle, Hypoxia, Tumor microenvironment, Cancer therapy, Tirapazamin

## Abstract

**Purpose:**

Tumor hypoxia is a key barrier to successful delivery and activity of anti-cancer agents. To tackle this, we designed hypoxia-responsive Au-PEI-Azo-mPEG nanoparticles (NPs) denoted as APAP NPs for targeted delivery of hypoxia-activated prodrug (HAP), tirapazamine (TPZ) to hypoxic breast cancer cells.

**Methods:**

AuNPs were first synthesized. And then, were coated with polyethylene imine (PEI) by EDC-NHS chemistry. To realize NP biocompatibility and self-activating potential, a hypoxia-cleavable mPEG-AZO linker shell was coupled to the Au-PEI core. The hypoxia-responsible behavior of nanoparticles was analyzed under 21% O_2_ (normoxia) and 1% O_2_ (hypoxia) condition in 2D cell culture as well as MDA-MB-231 and MCF-7 spheroids as reliable biomimetics of tumor hypoxia.

**Results:**

APAP NPs elicited comparable cytotoxicity upon MDA-MB-231 cancer cells lowering TPZ IC_50_ to 7.46 µg/mL after 24 h. And were capable of enhanced ROS generation (*P*<0.001), and reduced mitochondrial membrane potential under hypoxia condition compared to the control (*P*<0.0001). Further, these NPs induced widespread apoptosis in both 2D and 3D cancer cell culture (*P*<0.0001), significantly reduced cell adhesion density (*P*<0.01), increased cell uptake by ~100 folds under hypoxia condition, and destroyed large MCF-7 spheroids by 72 h.

**Conclusion:**

Together, APAP@TPZ as biocompatible, and multi-stage activating platforms afford deepened penetration of HAP to hypoxic tumor core, where PEG detachment and TPZ bioreduction into its active form promote selective and effective eradication of hypoxic breast cancer microtumors.

## Introduction

 Cancer is one of the main causes of death in the world. Currently, there are 17.2 million cancer patients worldwide, and 8.9 million of them die from cancer every year.^1^ Despite the remarkable progress achieved in recent years, no suitable and efficient method for prevention, diagnosis and effective treatment of cancer has been introduced yet.^2,3^ One of the most common cancer treatment methods is the removal of the primary tumor combined with chemotherapy, radiation therapy, and immunotherapy as routine treatment methods.^4-6^

 An additional solution for the treatment of cancer is to use nano drug delivery systems (NDDSs) with the aim to increase the amount of drug delivered to the tumor site, increase the efficiency of drugs and reduce toxicity.^7,8^ In one step forward, smart drug-carrying nanoparticles are introduced that can change their physico-chemical characteristics (size, surface charge, hydrophobicity, or hydrophilicity) in response to pathological changes in the tumor microenvironment (such as acidity, hypoxia, etc.).^9-13^ On the other hand, NPs design can affect the circulation, accumulation, permeability, cellular uptake and drug release, and thus the chance of nano drug formulations for clinical use.^7,14^

 Solid tumors are usually characterized by hypoxic and necrotic tissue, increased metabolic waste products that lead to a decrease in extracellular pH, and high intra-tissue hydrostatic pressure. Compared to normal tissues, the tumor microenvironment (TME) has special characteristics such as vascular abnormalities, hypoxia (oxygen deficiency conditions), hyperthermia, acidic pH as well as genetic/epigenetic changes in specific gene expression levels reflected in overexpression of specific tumor markers, e.g. matrix metalloproteinase enzymes.^15-18^ Using the characteristics of the TME in the design of nanoparticles leads to an increase in the efficiency of targeted treatment methods.^19^ Tumor hypoxia means the oxygen level in the tumor tissue is lower than the physiological oxygen levels. The oxygen pressure in some solid tumor tissues may be close to zero mm Hg, while in normal tissues this pressure is ~30 mm Hg.^16,20^ The rapid proliferation of tumor cells and the structural and functional abnormalities of the tumor limit the diffusion of oxygen. Access of cancer cells to oxygen also decreases with increasing distance from blood vessels. Therefore, hypoxic cells are far from blood vessels and thus out of reach of anticancer drugs to achieve effective treatment potential with conventional treatments.^21,22^ And even NDDSs. Other than that, it is well-known that tumor hypoxia is involved in tumor angiogenesis, proliferation, tumor metabolism which brings changes in low acidity, high temperature, and reductive sate as hallmarks of solid tumors.^23,24^ These conditions further promote cancer cell reprogramming and the emergence of cancer stem cells which contribute to invasion, metastasis, multi-drug resistance (MDR), and minimal residual diseases in cancer patients.^16,17,25-27^ On the bright side, tumor hypoxia can be used to control drug release or pro-drug activation in tumor tissue as attempted by the design of hypoxia-responsive NP formulations.^15,25,28-30^ Advanced generations of NDDSs can cross several biological barriers including escape from immune cells, tumor cell recognition, localization, accumulation, crossing cell membrane, and finally penetration and retention within cytoplasm of cancer cells by avoiding endosomal escape, proton pomp, and clearance.^4,24^

 Hypoxia-activated prodrugs (HAPs) are specifically transformed into the radical and active form of the drug in the hypoxic environment of cancer cells under the effect of the tumor-reducing environment. There are different drugs from this category that have been tested in different clinical phases such as TH302, tirapazamine (TPZ) and AQ4N. Among HAPs, TPZ and TH302 have undergone numerous preclinical and clinical studies (see for review ^16^). These drugs have high specificity for targeting hypoxic cells and their simultaneous use as a combination therapy along with other common methods such as chemotherapy or photodynamic therapy can be the most effective approach to hit the hard-to-reach tumor cells.^31-33^

 Azobenzene linker (azobenzene or AZO) has high specificity and sensitivity to hypoxia, as it is broken by NADPH-dependent azo reductase enzymes found abundantly in the TME of solid tumors due to reductive stress conditions in hypoxic cells. Smart polymer nanoparticles can be created by using specific linkers that respond to TME stimuli. In this regard, azo linkers can be used to crosslink different polymers together for the synthesis of hypoxia-responsive smart NPs.^34,35^ In this regard, core-shell NPs with self-activating potential can be designed where the core part composes of a small and high positive charge polymer such as chitosan,^36^ PAMAM dendrimer,^37^ or polyethylene imine (PEI) and shell part is a negative charge polymer such as poly ethylene glycol (PEG) which afford a negatively-charged stable nanoparticle. Core-shell parts are connected by tumor-specific bioreducible linker (e.g. azo linker). Examples of this class are cluster bombs with size/charge changing potential based on PAMAM where a large nanoparticle (~100 nm) adopts pH-responsive potential to localize specifically in tumor regions and then by cleaving pH-responsive linker, ultra-small size PAMAM (~10 nm) with a high positive charge can deliver and rapidly penetrate the cell membrane to deliver chemotherapeutic and inhibit lymph node metastasis in 4T1 mice.^38^

 Hypoxia is key for HAP activity, for TPZ is a non-toxic molecule, hypoxia reduces it to its active and toxic form (BTZ) through single electron reduction, and in the presence of oxygen, BTZ can revert to TPZ which diminish its activity. Given the critical role of hypoxia for optimum activity of HAPs, previous works have attempted to use strategies such as combining HAP with tumor infarction therapy (e.g. using CA4P as a vascular disrupting agent)^39^ or photodynamic therapy or radiotherapy which both consume O_2_ and thus aggravate hypoxia for better performance of HAP.^40-42^ Another strategy is the use of multi-stage acting NPs which can deliver TPZ to the hypoxic regions of tumor where it has the maximum activity.

**Figure 1:**
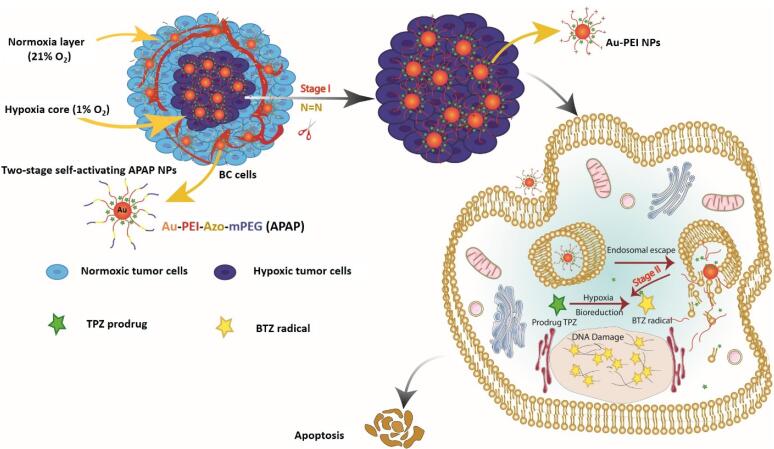
Design concept of APAP@TPZ NPs for selective cancer bioreductive therapy. Green asterisk indicates TPZ prodrug (inactive state) and yellow asterisk denotes activated TPZ (BTZ)

 With this in mind, we aimed to develop a simple yet effective hypoxia-responsive system to achieve self-activation for controlled, localized and efficient TPZ delivery and maximum activity in hypoxic tumor cells. Herein, we introduce a simple, biocompatible and efficient dual-stage acting smart nanoparticle capable of stepwise activation of nanoparticles for TPZ delivery into hypoxic TNBC microtumors and then TPZ activation into BTZ radicals (Figure 1). For this purpose, we coated the surface of Au-PEI core with a detachable shell of mPEG-Azo. In this design, firstly, the PEG layer increases the stability of the nanoparticle, and secondly, the separation of this layer under hypoxic environment increases the positive charge of the nanoparticle due to exposing PEI layer for deepened penetration into tumor hypoxic core. Meanwhile, AuNPs were used as core upon which PEI layer can be assembled and immobilized. Thirdly, under hypoxic condition HAP undergo bioreduction forming active drug to selectively eradicate hypoxic tumor cells embedded in the deep tumor layers. Thus, APAP NPs can selectively target hypoxic tumor cells in the highly heterogeneous microenvironment of triple-negative breast tumors.

## Materials and Methods

###  Materials 

 Bi-functional 4,4-dicarboxylic Azo linker (AZ) with 300 Da MW was purchased from TCI, USA. Methoxy PEG (NHS-PEG-methoxy, *M*_W_ 1000 Da), 1-Ethyl-3-[3-(dimethylamino) propyl] carbodiimide (EDC), N-hydroxysuccinimide (NHS), GSH, cellulose dialysis bag (cut off 12-14 kDa), 4,6-diamidino-2-phenylindole dihydrochloride (DAPI), 2′,7′-Dichlorofluorescin diacetate (H2DCFDA), phosphate buffered solution (PBS), 3-(4,5-dimethyl-2-thiazolyl)-2,5-diphenyl-2H-tetrazolium bromide (MTT), and Trypsin-EDTA, and linear polyethylenimine (PEI, C2H5Nn) MW 25000 Da), TPZ, doxorubicin and HAuCl4 salt were obtained from Sigma-Aldrich St. Louis, MO, USA. The source of other used materials was as follows: Annexin V Apoptosis Detection Kit (MabTag, Germany), RPMI1640 and FBS (AnnaCell, ScienCell, USA), Pensterp solution (P/S, ScienCell, USA), Rhodamin 123 (Dojindo, Japan). All other reagents and solvents used in this work were analytical grade and were obtained from Merck.

###  Synthesis of hypoxia-sensitive nanoparticle

####  Synthesis of AuNPs 

 The synthesis of AuNPs was based on our previous works.^14,43,44^ Briefly, 15 mL (0.3 mmol) of gold salt (HAuCl4) was dissolved in 5 mL distilled water on the hot stirrer till it reached the boiling temperature, at which point 0.4 mL of 1% (w/v) trisodium citrate was added. After 5 minutes, the reaction was continued on a cold stirrer till the reaction was complete. Then, 2.5 mg of mercaptodecanoic acid (MUA) in 1 mL ethanol was added to the as-prepared solution. Afterward, pH was adjusted to 8, and the reaction was purified with a 10 kDa dialysis bag. The dialysis medium was refreshed every 8 h and pH was kept on 8. After 48 h, samples were removed from dialysis bags and stored at 4 °C for the next experiment.

####  Synthesis of mPEG-Azo copolymer

 mPEG-Azo copolymer was synthesized according to the protocol described by Xie, Z et al ^35^ with some modifications. For this, 500 mg (0.5 mmol) of mPEG was added to 200 mg (0.67 mmol) of azolinker. This reaction was carried out in the presence of EDC (140 mg, 0.7 mmol)/NHS (80 mg, 0.7 mmol), and pyridine as solvent at room temperature for 24 h. Then the product was dissolved in dichloromethane and precipitated in cold diethyl ether. After the purification of the product, the synthesis of mPEG-Azo-linker was confirmed by FTIR.

####  Synthesis of Au-PEI-Azo-mPEG (APAP) NPs

 First, 17.3 mg (0.09 mmol) of EDC and 10 mg (0.09 mmol) of NHS were added to 5 mL of as-synthetized gold nanoparticle and mixed for 1 h. Then, 50 mg (0.002 mmol) linear PEI (*M*_W_: 25000 Da) dissolved in 5 mL deionized water was dropwise added to the solution and left on the stirrer for 24 h. Later, 30 mg of activated mPEG-Azo with EDC and NHS was added to the solution containing Au-PEI NPs on the stirrer. After 24 h, the mixture was dialyzed (10 kDa cut off) for two days. And then centrifuged at 3000 rpm for 10 min to remove remaining impurities. The final product was freeze-dried and characterized by SEM, FTIR, and Zeta sizer.

####  Drug loading and release 

 To prepare APAP@TPZ NPs, 1 mg TPZ dissolved in 1 mL ethanol was added to 1 mL APAP NPs solution on a magnetic stirrer for 24 h at room temperature. Next, the reaction was centrifuged at 3,000 rpm speed for 10 min and washed 3 times with PBS to remove unencapsulated TPZ. The supernatant (1 mL) was then poured into a dialysis bag (MWCO = 12 kDa) and sunk in 25 mL PBS (pH = 7.3) at 4 °C on a magnetic stirrer for 0.5 h. Then, the amount of released TPZ was detected by UV-vis spectrometry (Cecil BioAquarius CE 7250, England) at the excitation wavelength of 470 nm. Drug encapsulation efficiency (DEE) was calculated by this equation:


(1) $$EE=\frac{Amount\;of\;drug\;loaded}{Initial\;amount\;of\;drug\;added}\times 100$$

To determine the drug release behavior of NPs, APAP@TPZ suspension (1 mg/mL) was poured into cellulose membrane tube (12 kDa MWCO) and dialyzed against PBS buffer (25 mL) at pH 7.3 and 5.4 at 37 °C temperature in a shaker incubator (150 rpm) with and without the addition of 10 mM GSH. At specified time intervals, 1 mL aliquots of samples were removed and replaced with fresh preheated medium to maintain a constant volume. The concentration of TPZ was measured using a UV-vis spectrophotometer (Cecil BioAquarius CE 7250, England) and used to calculate cumulative drug release.

###  2D cell culture 

 To perform MTT assay, MDA-MB-231 cells were cultured at 37 °C, 5% CO_2_ under 1% O_2_ or 21% O_2_ to mimic hypoxic and normoxia conditions, respectively. After reaching 80% cell confluency, the cells were harvested and counted with trypan blue staining and 1 × 10^4^ cells were seeded to each well of 96 well plate and placed into incubator for 24 h to allow cell attachment. Following, treatments for the 4 groups, including the control group, APAP NPs, APAP@TPZ, and free TPZ were conducted in triplicates. Then, plate was incubated for 24 h and MTT solution with a concentration of 0.5 mg/mL was added to each well. MTT dye is absorbed by the mitochondria of living cells and is converted into formazan crystals under the influence of enzymes in the mitochondria. Next, the plate was incubated for 4 h until purple formazan crystals were formed. Formazan crystals were dissolved in 100 µL DMSO. Finally, the absorbance was measured by the ELISA device at a wavelength of 570 nm (Stat Fax 4200, RayBiotech INC, USA). Viability is calculated from the following equation: %viability = (mean OD sample/ mean OD control) × 100. Also, a hypoxia incubator with 1% oxygen was used to imitate the hypoxia environment. To this, after 24 h cell seeding into 96 well plate, the cells were incubated for 6 h in a hypoxia incubator, and then drug treatment was performed with the same doses similar to normoxia conditions for nanoparticles loaded with TPZ and free TPZ drug. After 24 h, cytotoxicity was checked by MTT method.

###  3D cell culture

 According to our previous works, we used MCF-7 and MDA-MB-231 mamospheres to form TME hypoxia biomimetics.^36,45^ To this, after cell culture and reaching 70% confluence, the cells were trypsinized and counted by trypan blue staining, and 2 × 10^5^ cells were added to 1% agarose gel pre-coated 24-well plates. Spheroids with > 300 μm size were used for further studies.

###  DAPI-collagen staining

 F-actin/DAPI dual staining was used to check the effect of APAP@TPZ on cell density.^46^ For this, 5,000 cells were seeded in 96 well plates and treated with nanoparticles for 24 h under normoxia and hypoxia conditions. Then the treated cells were fixed in 4% paraformaldehyde, washed three times with PBS, and incubated with 0.1% Triton X-100 for 10 minutes for permeability. After that, cells were washed three times with PBS and stained with phallacidin for 45 minutes and DAPI dye for 5 minutes. Results were imaged by Cytation^TM^ 5 cell imaging instrument (BioTek, Winooski, USA).

###  Nanoparticle cellular uptake 

 APAP@DOX (1 µg/mL) was used to investigate the cellular uptake of nanoparticles in MDA-MB-231 tumor cell cultured under normoxia and hypoxia conditions.^47,48^ Accordingly, 4 h after treatment with DOX or APAP@DOX, the cells were trypisnized, centrifuged with 1500 rpm for 5 min and washed with PBS twice and then were analyzed by flow cytometry instrument (BD FACS Caliber, Becton Dickinson, San Jose, CA, USA) with an excitation wavelength of 570 nm for doxorubicin.

###  Rhodamine 123 (Rh123) staining

 According to our previous works,^36,37^ MDA-MB-231 cells were cultured in hypoxia and normoxia conditions and then treated with APAP@TPZ20 for 24 h. The supernatant was discarded and the cells were washed three times with PBS (pH 7.3) and mixed with 5 μg/mL Rhodamine-123 at 37 °C for 15 min. Then the cells were washed with PBS and the plates were immediately scanned by the cell imaging system (Cytation^TM^ 5 BioTek, Winooski, USA).

###  DCFH staining

 DCFH staining was performed According to our previous works,^36,37^ Briefly, MDA-MB-231 cells cultured under 1% hypoxia and 21% normoxia in 24-well plates were treated with APAP@TPZ20 for 24 h. The next day, the desired cells were treated with a 10 μM H2DCFDA in RPMI-1640 medium for 24 h at 37 °C. Next, the supernatant was discarded and the cells were washed 3 times with PBS. Finally, the reduction of DCF substance and the fluorescence intensity was immediately read at wavelengths of 475-570 nm by a microplate reader (BioTek, Winooski, VT, USA).

###  Annexin V-FITC/PI staining

 Annexin V-FITC/PI staining was used to determine the amount of live (pre-apoptotic) cells and dead (apoptotic) cells, respectively, under 2D and 3D cell culture. To this, 24 h after treatment with APAP@TPZ NPs, cells were washed by PBS twice and then stained with 5 µL of Annexin V-FITC and 2 μL of PI dye for 10 minutes. Results were analyzed using Citation V cell imaging system with filters of 515-545 nm for FITC and 535-617 nm for PI.

###  Statistical analysis

 Data analysis was done by GraphPad Prism 9.0 software. One-way or Two-way ANOVA and Tukey’s post hoc test were used to perform mean difference tests. A statistically significant level of *P* < 0.05 was considered. Images were quantified using Image J software.

## Results

###  Synthesis and characterization of APAP NPs

**Figure 2:**
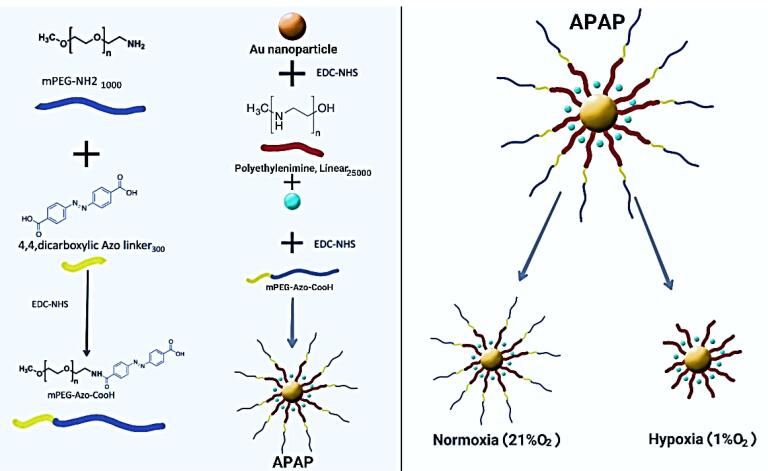
Concept and working principle of APAP NPs

**Figure 3:**
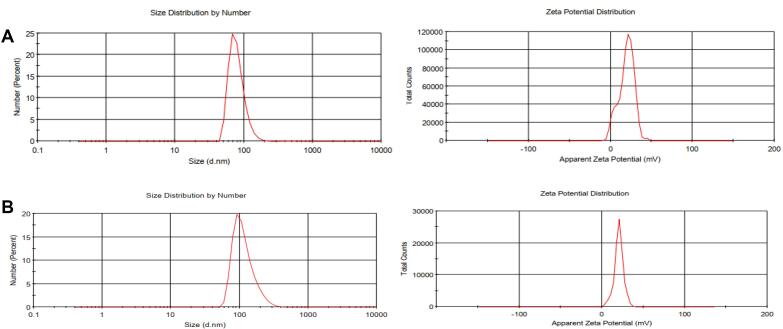
Size and zeta potential of synthesized NPs. **a,** APAP, **b**, APAP@TPZ. TPZ loading increased the size of NP, producing final NPs with ~116.9 nm

 The schematic process for NP synthesis is shown in Figure 2. Nanoparticle characterization was analyzed using DLS, FTIR, and SEM microscopy. DLS analysis showed an increase in the size and surface charge of AuNPs following PEI modification which increased zeta potential from -11.7 mV to + 27.8 mV which was suitable for cellular uptake. After pegylation, zeta was reduced to + 19 mV and the final particle size was ~79.76 nm (Table 1 and Figure 3).

**Table 1:** Characteristics of synthesized nanoparticles.

**NP**	**Size (nm)**	**PDI**	**Zeta potential (mV)**
AuNPs	52.90 ± 14.7	0.171	-11.7
Au-PEI	74.98 ± 1.94	0.33	27.8
Au-PEI-Azo-mPEG	79.76 ± 21.85	0.34	19.5
Au-PEI-Azo-mPEG@TPZ	116.9 ± 45.3	0.317	15.6

**Figure 4:**
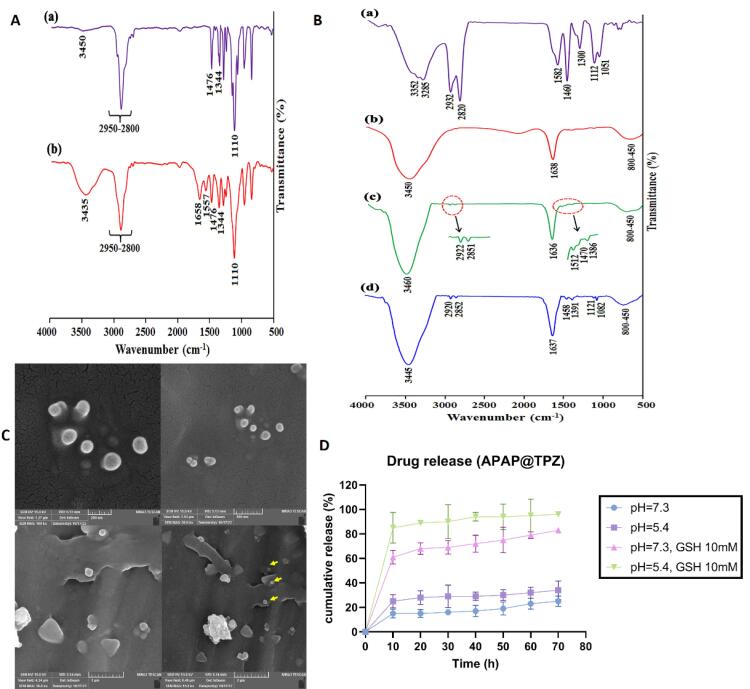
Characterization of nanoparticles. **A**, The FTIR spectra of mPEG (a), mPEG-Azo (b). **B**, The FTIR spectra of PEI (a), AuNPs (b), Au/PEI (c), Au/PEI-g-mPEG NPs (d). **C**, Morphology of APAP@TPZ NPs under SEM microscope. The results of SEM images were in line with DLS results, confirming the generation of APAP@TPZ NPs with desirable size (~120 nm) and spherical morphology to serve as NDDSs. Nanoparticle is shown with different scales. **D**, Drug release profile of APAP@TPZ NPs. Drug release of TPZ was significantly enhanced in acidic pH in the presence of GSH compared to acidic pH without GSH

 The FTIR spectrum of the mPEG exhibited the stretching vibration of terminal hydroxyl group as a broad and weak band at 3450 cm^-1^, the stretching vibrations of aliphatic C—H groups related to the PEG backbone at 2950 to 2800 cm^-1^ region, the bending vibrations of C—H groups at 1344 and 1467 cm^-1^, and the stretching vibration of C—O group as a strong band at 1110 cm^-1^ (Figure 4Aa). The successful functionalization of mPEG with azobenzene-4,4’-dicarboxilic acid moiety was verified by the appearance of some new adsorption bands, including the stretching vibration of aromatic C = C at 1557 cm^-1^ and the stretching vibration of carboxyl group at 1658 cm^-1^ (Figure 4Ab).

 The FTIR spectrum of the PEI (Figure 4Ba) showed the stretching vibrations of various C—N groups at 1300, 1112, 1051 cm^-1^, the bending vibration of C—H group at 1460 cm^-1^, the bending vibration of N—H group at 1582 cm^-1^, the stretching vibrations of C—H groups at 2932 and 2820 cm^-1^, and the stretching vibrations of N—H groups at 3352 and 3285 cm^-1^. The most important adsorption bands in the FTIR spectrum of the AuNPs are the stretching vibration of carboxylate group at 1638 cm^-1^ and the stretching vibration of surface hydroxyl groups at 3450 cm^-1^ ^49^ (Figure 4Bb).

 The appearance of some new adsorption bands in the FTIR spectrum of the Au/PEI NPs, including the stretching vibration of C—N group at 1386 cm^-1^, the bending vibration of C—H group at 1460 cm^-1^, the bending vibration of N—H group at 1512 cm^-1^, and the stretching vibrations of C—H groups at 2922 and 2851 cm^-1^ confirm the chemical coating of AuNPs with PEI chains (Figure 4Bc). Finally, appearance of the stretching vibrations of C—O groups (related to mPEG backbone) at 1082 and 1121 cm^-1^ revealed the successful synthesis of Au/PEI-*g*-mPEG (Figure 4Bd).

 Further, examining nanoparticles with an SEM microscope revealed the synthesis of APAP@TPZ nanoparticles with spherical morphology and nano size ( < 150 nm) (Figure 4C). Also, the drug release profile of NPs was determined at 25% and 34% in the conditions of the physiological environment (blood) with pH = 7.3 and the acidic environment simulating the TME, respectively, while in the conditions of simulated hypoxia (GSH as reductive agent) and acidic pH, ~86% of the drug was released within 72 hours (Figure 4D).

###  Cytotoxicity assay

**Figure 5:**
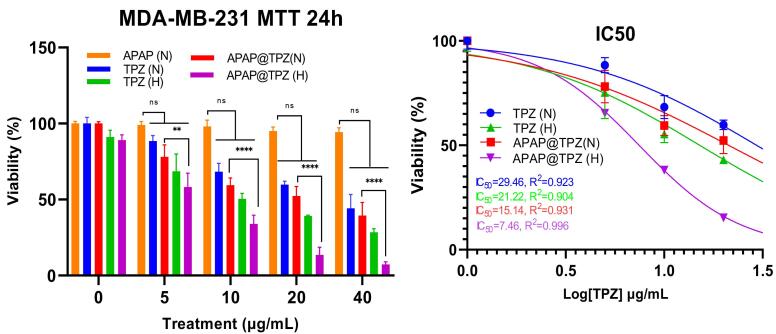
MTT confirmed biocompatibility of APAP NPs while potent cytotoxicity of APAP@TPZ under hypoxia conditions. The IC50 of free TPZ was 21.22 µg/mL in hypoxia condition compared to APAP@TPZ with IC50 of 7.46 µg/mL. Two-way ANOVA-Tukey Post HOC test, ns: none significant, *P < 0.05 **P < 0.01, ***P < 0.001, ****P < 0001

 MTT test was used to check the cytotoxicity of synthesized nanoparticles and the viability of MDA-MB-231 cells treated with nanoparticles under normoxia and hypoxia conditions. The results showed that in both hypoxia and normoxia conditions, the synthesized nanoparticles have no significant toxicity on the studied cancer cell line at any given concentrations, confirming the biocompatibility of APAP NPs (Figure 5). Meanwhile, APAP@TPZ showed superior dose-dependent cytotoxicity *vs.* free TPZ drug at all concentrations. The efficacy of free TPZ and APAP@TPZ in hypoxia condition was significantly enhanced compared to normoxia conditions at any given dose. Under hypoxia conditions, the IC_50_ values of free TPZ and APAP@TPZ were 15.14 μg/mL and 7.46 μg/mL, respectively. Meanwhile, under normoxia condition, IC_50_ value of free TPZ and APAP@TPZ was determined 29.46 μg/mL and 21.22 μg/mL respectively. IC_50_ was not calculated for APAP, as there was no observable dose-dependent cytotoxicity in this group. Also, in normoxia conditions, no significant difference was observed between the free TPZ drug and APAP@TPZ at any dose. Furthermore, APAP@TPZ was also tested on MCF-7-10A as normal breast cancer cell line. There was more than 90% viability at all tested concentrations (0, 5,10, 20, 40, 50 μg/mL), which further confirms the high biocompatibility and selectivity of designed NPs for cancer cell targeting (Figure S1).

###  The effect of APAP@TPZ on cell adhesion density

**Figure 6:**
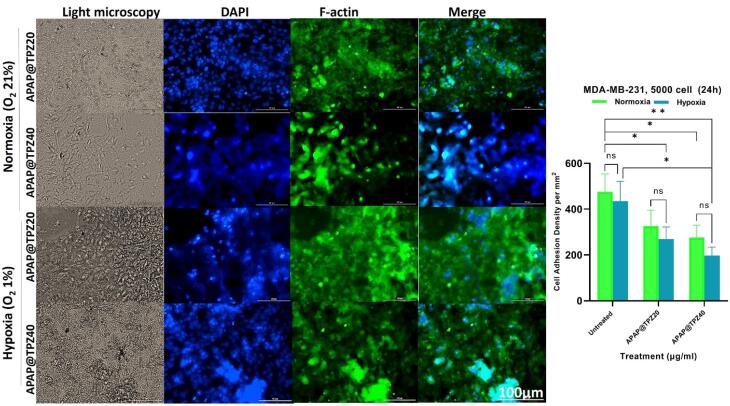
The effect of APAP@TPZ on cell adhesion density. Under hypoxia, APAP@TPZ40 significantly reduced cell adhesion density under hypoxia conditions compared to the untreated group. Two-way ANOVA-Tukey Post HOC test, ns: none significant, *P < 0.05, **P < 0.01, ***P < 0.001, ****P < 0001

 DAPI/F-actin staining was used to determine the effect of hypoxia-sensitive nanoparticles on cell adhesion density.^46^ APAP@TPZ resulted in decreased cell adhesion density in a dose-dependent manner compared to the untreated controls, and these changes were marked under hypoxia compared to the normoxic conditions (Figure 6).

###  ROS activity

**Figure 7:**
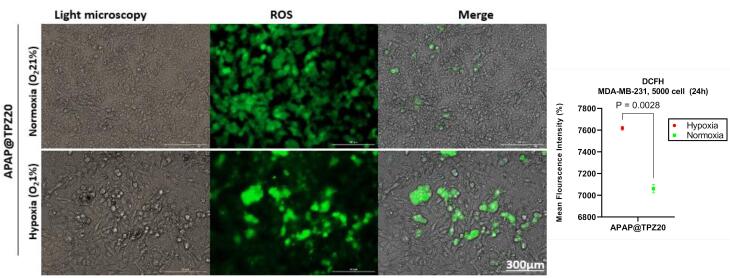
The effect of APAP@TPZ on ROS level in MDA-MB-231 cancer cells under normoxia and hypoxia conditions. APAP@TPZ20 significantly promoted ROS generation under 2D cultured hypoxia compared to normoxia conditions. Unpaired t test

 DCFH staining was used to measure the amount of intracellular reactive oxygen species (ROS) under normoxia and hypoxia conditions using 2-dichlorofluorescein diacetate (H2DCFDA) as a molecular fluorometric probe for measuring ROS and oxidative stress.^37^ Oxidation of DCF by mitochondrial enzymes and in the presence of oxygen free radicals including peroxynitrite, hydroxyl peroxidase causes the production of a very strong fluorescent color, the intensity of which is directly related to the amount of intracellular ROS. Data from our previous work confirmed that in untreated controls, ROS generation was significantly higher in hypoxia (6698.971 ± 17.345) compared to normoxia (5583.607 ± 15.453) cell culture conditions.^36^ We observed a significant increase in ROS level in 2D cultured cancer cells treated with APAP@TPZ2 under hypoxia (7617.556 ± 16.85) *vs.* normoxia (7060.556 ± 38.571) condition *in vitro* (Figure 7).

###  Mitochondrial membrane potential 

**Figure 8:**
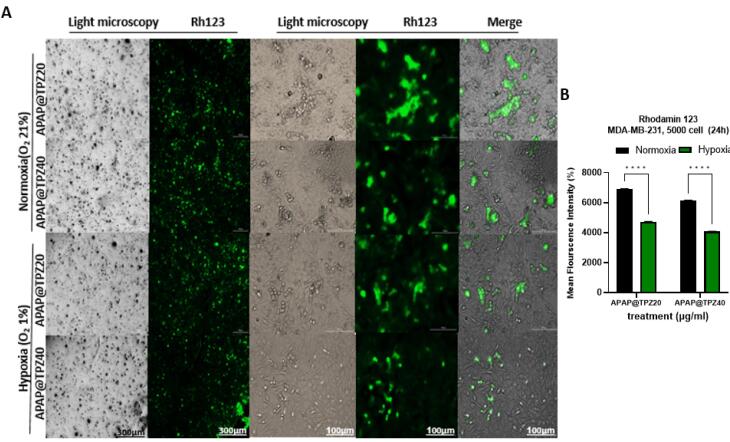
APAP@TPZ significantly reduced mitochondrial membrane potential under hypoxia *vs.* normoxia conditions, which can be explained by cleavage of the hypoxia-responsive azo linker and exposure of cationic PEI-gold core, and then TPZ delivery and activation to BTZ toxic radicals under hypoxia condition. Two-way ANOVA-Tukey Post HOC test, ****P < 0001

 We next studied changes in mitochondrial membrane potential (∆ψm) by Rhodamine 123 staining in cancer cells treated with APAP@TPZ under normoxia and hypoxia conditions. Rhodamine 123 is a fluorescent cationic lipophilic dye that can bind to metabolically active mitochondria. As a result, the activity of the cell is directly related to the intensity of the fluorescent color.^36^ Our results showed that in the disturbed cells under the influence of hypoxic stress, which were treated with two different doses of TPZ-loaded nanoparticles, the intensity of the color compared to the cells with normal metabolic activity (normoxia conditions) was significantly reduced (Figure 8).

###  Determining the live/dead cells in 2D cell culture

**Figure 9:**
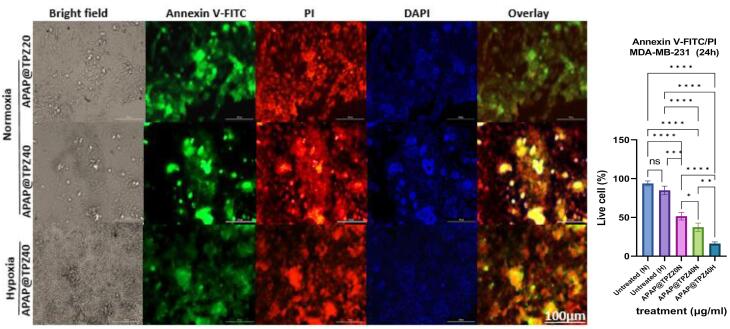
Live-dead staining of APAP@TPZ-treated cells under normoxia and hypoxia conditions in 2D cell culture. APAP@TPZ had superior induction of apoptosis under hypoxia condition, which shows the selective activity of formulated NPs against hypoxic tumors. Two-way ANOVA-Tukey Post HOC test, ns: none significant, *P < 0.05, **P < 0.01, ***P < 0.001, ****P < 0001

 The anti-cancer potential of APAP@TPZ was evaluated using live/dead staining in 2D cultured cancer cells under normoxia and hypoxia incubator for 24 h. There was no significant difference in the viability of cultured controls under both conditions, meanwhile in tumor cells treated with different doses of APAP@TPZ, dose 40 µg/mL (APAP@TPZ0) reduced the percent of viable cancer cells to 16.267 ± 2.2% (hypoxia) and 37.407 ± 5.3% (normoxia) compared to untreated control with 84.943% (hypoxia condition) and 93.813 ± 3.2% (normoxia) (Figure 9).

###  Live-dead staining in the spheroid model

**Figure 10:**
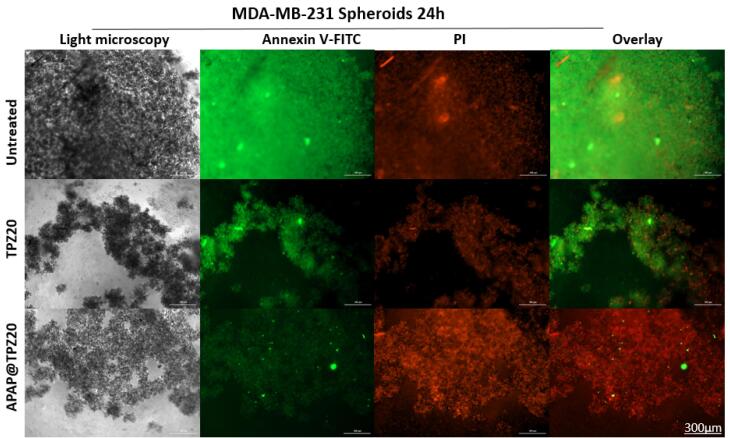
The effectiveness of hypoxia-sensitive nanoparticles in inducing apoptosis after 24 h in breast cancer spheroid model. The green color (annexin-FITC) and red color (pi) stain live and dead cells, respectively. Treatment with APAP@TPZ20 showed widespread induction of apoptosis as well as penetration of nanoparticles into the hypoxic regions in TNBC large spheroids. Free TPZ drug, on the other hand, shows a partial cell-killing effect due to its low penetration into the hypoxic regions, where it can undergo single electron reduction to form tumor-killing BTZ toxic radicals

 Using 3D cultured mamospheres, we studied the efficacy of APAP@TPZ for penetration and induction of apoptosis by annexin-FITC/PI staining. Our results indicated the superior performance of APAP@TPZ in the spheroid model *vs *2D cell culture as a reliable model for studying tumor hypoxia. We observed almost 100% induction of apoptotic death in APAP@TPZ treated cancer spheroids compared to the untreated control with ~100% viable cancer cells and incomplete induction of cell apoptosis ( < 50%) in free TPZ-treated cancer cells (Figure 10).

###  Cellular uptake of nanoparticles

**Figure 11:**
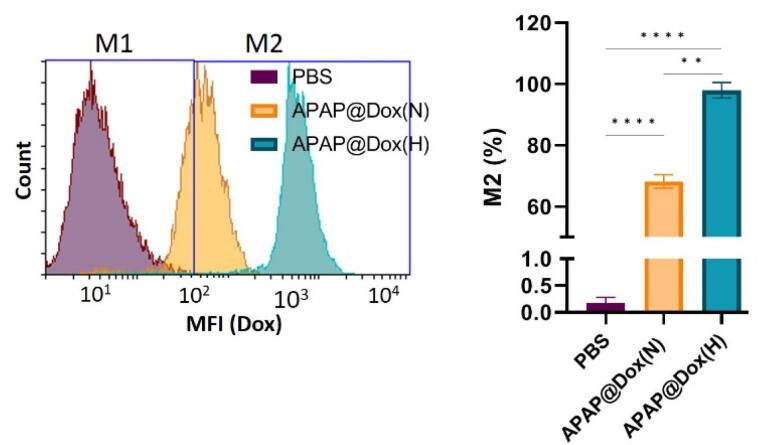
Cellular uptake of APAP@DOX NPs. Hypoxia promoted selective uptake of APAP NPs compared to normoxia condition by a ~100 folds increase in MFI possibly due to detachment of PEG-Azo hypoxia crosslinker, which produces cationic PEI-gold with positive charge ( + 27.8 mV) and small size (74 nm) to swiftly penetrate and cross cancer cell membrane for TPZ delivery

 Given the inherent fluorescent properties of doxorubicin (Dox), we used APAP@Dox nanoparticles to study NP uptake using flowcytometry, which indicated enhanced NPs uptake by ~1.4 fold and ~100 folds increase in MFI under hypoxia conditions *vs* normoxia possibly due to the detachment of PEG shell and exposure of cationic PEI core (Figure 11).

###  The effect of APAP@TPZ on MCF-7 tumor spheroids

**Figure 12:**
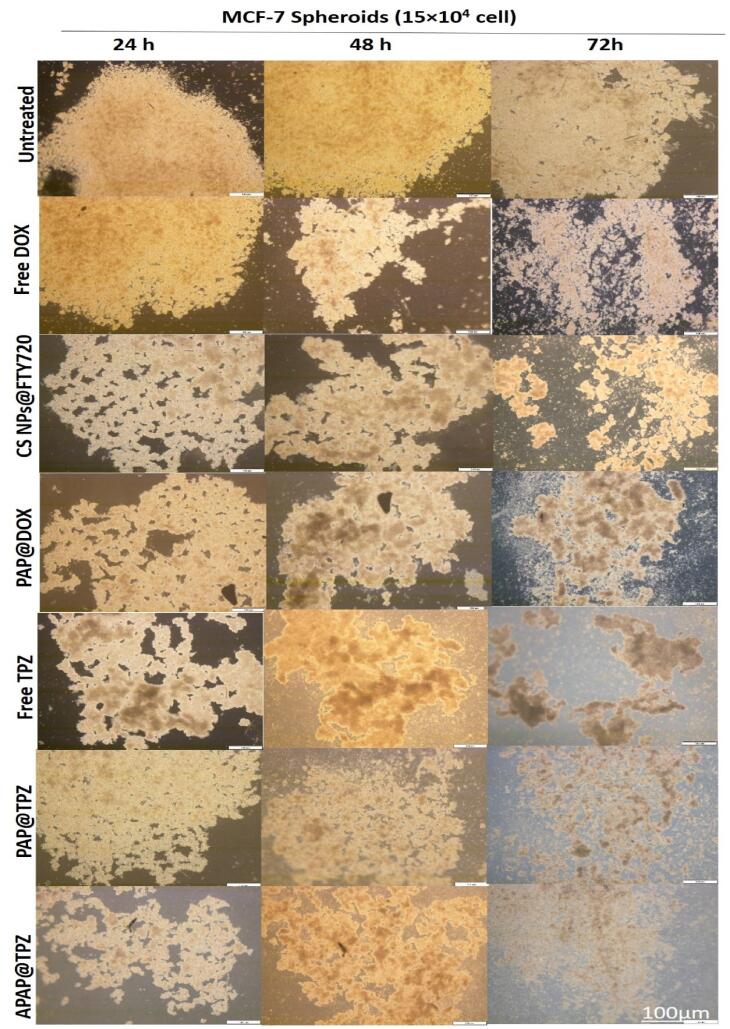
The effect of different hypoxia-responsive NPs on large hypoxic breast cancer spheroids. APAP@TPZ is capable of widespread and homogenous penetration into all tumor regions, including hypoxic layers for maximized TPZ activity

 In line with our previous experiments,^36,37^ we further studied the effect of different formulated hypoxia-responsive NPs, including PAMAM-Azo-mPEG (PAP)^37^ and chitosan-Azo-mPEG (Cs NPs)^36^ along with APAP in terms of their mode of penetration and anti-cancer efficacy on breast microtumors to mimic TME hypoxia. We find that free drugs such as Dox were mostly efficient on the tumor edges, starting to eat the tumor from the outside, and reaching maximum effect by 72 h. Unlike DOX, treatment with free TPZ generated spongy-like structure, which is plausible by TPZ optimum activity upon hypoxic tumor cells, which resulted in disintegration of microtumor by 72 h. Among hypoxia-responsive polymeric nanoparticle formulations, chitosan and PAMAM G5 dendrimer as cationic core NPs resulted in homogenous distribution of target drugs (TPZ, DOX and FTY720). PAP@TPZ and APAP@TPZ were capable of complete disintegration of tumor spheroids by 72 h. On the other hand, the most appealing results was for APAP@TPZ with cationic PEI-Au core which resulted in almost complete destruction of microtumors, owing to the depth and homogenous distribution of PEI-gold NPs within whole tumor region which combined with maximum activity of TPZ to destroy hypoxic cancer cells (Figure 12).

## Discussion

 The history of nanoparticles for cancer treatment starts from a simple liposome containing doxorubicin and continues to complex bioreactive, multifunctional and intelligent engineered nanoparticles. However, until now, a preferred nanoparticle has not been able to eradicate cancer due to several obstacles including selective accumulation at the tumor site, inconsistent drug release, lack of optimal conditions for the activity of the released drugs, and uptake of nanoparticles by non-cancerous cells.^25,47,50^ Therefore, to pass through biological barriers, smart nanoparticles are required to be able to adapt to the environmental conditions in which they are placed and to provide the targeted delivery of a specific dose of medicine to the tumor site with minimal side effects.^26,51,52^ One of these strategies is the design of smart polymer nanocarriers that are able to respond to external stimuli such as temperature or take advantage of tumor pathological conditions such as acidity for specific activation in the TME.^53-55^ One of the most advanced forms of smart nanoparticles is those that are able to change physicochemical properties such as surface charge and size in response to the presence of specific stimuli. Such that, NP design consists of a core with a positive charge such as chitosan, PEI, or dendrimer and a shell usually made of polyethylene glycol, which can be broken by a linker sensitive to external or internal stimuli. The decomposition of a large nanoparticle with a negative surface charge produces small-sized NPs with a positive charge, which can be swiftly picked up by cancer cells. Meanwhile, such nanoparticles are devoid of binding to opsonins and protein corona formation, thus they possess long circulation time and their absorption and capture by non-target cells and organs can be prevented.^41,42^ Furthermore, cationic polymers not only promote enhanced drug uptake due to the presence of high amin groups, but they also promote endosomal escape of NPs and drug delivery through the proton sponge effect, which further augment NP accumulation and retention inside the target cells to achieve therapeutic action of chemotherapeutic drugs.^4,26^

 A recently popular Trojan system resembles a cluster bomb or charged jet bomb, assembled through the reversible cross-linking of thousands to millions of small particles that form a larger particle. The larger size (more than 100 nm) allows the selective and specific accumulation of the nanobomb in the tumor using the EPR effect. Later, to enable uniform distribution throughout the tumor region, the size/charge modality is activated to break down the nanobomb into small (positively) charged particles with high permeability to perforate the extracellular matrix. ^51^ Nanobombs vary in response to stimuli and can change their charge/size to internal stimuli such as acidity, oxygen, redox, and tumor-specific proteases (e.g., matrix metalloprotease, hyaluronidase) or external stimuli (light, ultrasound, and magnetic fields) or a combination of these stimuli.^56,57^

 Another example where changing the size of a nanoparticle allowed it to penetrate deeper into tumor regions involved a 100 nm iCluster polymer cluster nanoparticle linked to a larger nanoparticle by a pH-sensitive amide linker (PCL-CDM-PAMAM/Pt). When accumulated at tumor sites, the lower pH of the TME triggers the release of ∼5 nm diameter poly(amidoamine) PAMAM dendrimers loaded with platinum prodrug, which enables both cellular internalization and tumor penetration. The high capability of this platform to treat various tumors such as metastatic cancer, drug-resistant cancer and pancreatic cancer with poor permeability in the body was confirmed.^41^ In another study, icluster has been reported to inhibit lymphatic metastasis and complete eradication of breast cancer tumors lasting more than 120 days in the 4T1 mouse model.^38^

 Taking advantage of the transfecting ability of PEI, a resizable polymeric micelle system mPEG-PLA-ss-PEI-DMMA is reported for nucleus delivery of payloads. More specifically, the NP has a double shell that increases in size under acidic pH conditions and changes into smaller micelles in the presence of intracellular glutathione. This system is able to deliver anticancer drugs directly into the nucleus of MDR tumor cells to effectively combat drug-resistant breast cancer. At the same time, for micelles with no size-changing ability, nanoparticles accumulate more in the cytoplasm than in the nucleus.^51^

 In line with our study, in addition to acid-sensitive linkers, hypoxia-sensitive linkers have also been used in the manufacture of smart nanoparticles for HAP delivery. For example, dual hypoxia-responsive nanocarrier including polyethylene-alkylnitroimidazole (PA)/hyaluronic acid(HA)-chlorine ce6 loaded with TPZ is reported for HA-mediated tumor-specific delivery and combined photodynamic chemotherapy. With this in mind that Combretastatin A4 (CA4) drug can cause irreversible occlusion of tumor vessels, another study employed intravascular injection of poly(l-glutamic acid)-graft mPEG@CA4 combined with intraperitoneal injection of TPZ. This hypoxia-activated therapeutic strategy specifically exacerbates tumor hypoxia and increases the efficacy of conventional HAP therapy against 4T1 metastatic breast cancer and affords complete tumor eradication (40 + 48 mg kg-1 on CA4 basis).^39^

 In another study, high-conversion nanoparticles (PEG-UCNP) are used to enhance the permeability of photosensitizer (PS) to induce near-infrared (NIR)-dependent “on-demand” release of AQ4N encapsulated from an amphiphilic diblock copolymer (PNBOC) polymersome. NIR activation of the hypoxia-sensitive functional group, 2-nitrobenzyl, changes the hydrophobic to hydrophilic state, which converts the polymersome membrane from impermeable to permeable. The higher permeability of PS and the sustained release of AQ4N up to 24 h later allow for combined chemophotodynamic therapy.^58^

 One of the main goals of using nanomedicines is localizing the effects of drugs while reducing their toxicity. In this regard, nanoparticles can be conjugated with specific ligands that are specific to receptors expressed in tumor cells, or smart nanoparticles with the ability to self-activate in the microenvironment specific to cancer tumors can be designed.^7^ In this regard, the present study set the proof of principle for development of two-stage acting hypoxia-sensitive nanoparticles, with ability to function specifically in the hypoxic microenvironment of solid tumors and deepened delivery of HAP drug into hypoxic tumor region, where it can reduce into the active radical forms to elicit maximum toxicity. In addition to the APAP NPs, we have designed other hypoxia-sensitive NPs based on chitosan^36^ and PAMAM G5.^37^ We confirmed tumor hypoxia self-activating nanoparticles are capable of specific localization and targeted and effective transfer of a range of cancer drugs to the deep layers of recapitulated solid tumors.^36^ We found that TPZ prodrug elicited some degree of toxicity in normoxia conditions as well, but its toxicity significantly enhanced under hypoxic environment in both 2D and 3D cell culture, likely due to decomposition of mPEG-Azo linker and exposure of Au-PEI cationic core NPs to drill into the hypoxic tumor cell membranes. Similar to our work, one study, used 4,4, dicarboxylic azobenze for synthesis TAT-Azo-PEG-Azo-PLGA for TPZ and CE6 delivery as a stepwise-activatable hypoxia triggered nanocarrier-based for photodynamic therapy and effective synergistic bioreductive chemotherapy. In this study, authors indicated no change in cell viability under normoxia condition. Also, they employed 2% oxygen as hypoxia condition. The IC50 of TAT-azo-NPs under acidic pH (6.8) and 2% hypoxia after 72h was ~ 2 µg/mL while for TAT-AZO-NPs @TPZ/CE6 at pH = 7.4, IC50 was approximately 5 µg/mL.^40^ Other formulation synthetized alkylated 2-nitroimidazole (ANI)-modified PEI (PA) and Ce6-grafted hyaluronic acid (HA-Ce6) as amphiphilic polymers capable of self-assembly into NPs to encapsulate TPZ. The IC50 free TPZ and PA/HA-Ce6@TPZ NPs after 72h on 4T1 breast cancer cells were ~ 5 µg/mL and < 0.5 µg/mL, respectively. In stimulated hypoxia condition (100µM Cocl2), the IC50 of NP was 0.4 vs > 4 µg/mL in normoxia condition.^42^ In our study, we used 1% oxygen (hypoxia incubator) and the IC50 of APAP@TPZ on MDA-MB-231 cancer cells after 24 h was 7.46 µg/mL which shows high potential of gold-PEI for possible directed delivery of TPZ into the cell nucleus where it produces oxidizing radical species.^16^

 One of the main challenges in this study, as well as other similar studies, is determining the amount and intensity of hypoxia, and before conducting studies related to nanoparticles and activated drugs, it is necessary to ensure the creation of hypoxia conditions similar to solid tumors.^16,59^ Two-dimensional *in vitro* culture fails to resemble the three-dimensional environment of solid tumors. On the other hand, in mouse tumor models, to check the efficiency of hypoxia-sensitive nanoparticle, the level of hypoxia in the tumor needs to be measured. Also, determining the severity of hypoxia in the tumor in clinical models that are candidates for HAP therapy is very important, as well as predicting the response to HAPs treatment in cancer patients. Fortunately, three-dimensional models of tumor spheroid with a size of 300 µm-1 mm established in the present study, were capable of inducing a hypoxia gradient similar to human solid tumors, yielding more reliable results of HAP therapy using hypoxia activating APAP NPs compared to 2D culture counterparts. In line with previous works, we find that 2D cell culture mimicking hypoxia does not fully resemble conditions of heterogenous hypoxia, instead anti-cancer action of hypoxic-responsive NPs, as normoxia cell culture itself can cause level of hypoxia and cellular stress which undermine the correct comparison of two states. For example, one study by folic-acid conjugated hypoxia-responsive glycol chitosan showed 20% increase in Doxorubicin toxicity on A549 cancer cells in hypoxia compared to normoxia condition, while for free DOX and MCF-7 there were no difference between in cytotoxicity among two conditions.^60^ It is documented that 3D cultured breast cancer (spheroids) with size more than 200 µm diameter can replicate,^61^ and thus are better mimetics of tumor hypoxia found in TME. In line with our previous works, we find MDA-MB-231 and MCF-7 mamospheres very helpful in providing a better estimation of hypoxia-responsive action of NPs. In one study, PEG-Azo-PAMAM was used for deepened delivery of HIF-1a siRNA and DOX. Dox fluorescence was apparent throughout MCF-7 spheroids, which indicated homogenous penetration of NPs in hypoxic spheroids.^35^

 Different types of HAPs, their structures, clinical stage as well as different nanoformulations are reviewed in detail in our previous work^16^ TH302 and TPZ are among the most studied HAPs in the preclinical and clinical settings. TH302 works under extreme hypoxia condition, meanwhile, TPZ shows some level of toxicity under normoxia condition, which can be explained by the constraints of 2D cell culture condition compared to the 3D cell culture. The main problem with HAP drugs is their high toxicity, which despite testing in many clinical phases, none entered into the market. In this formulation, we confirmed that the drug formulation with TPZ alone was able to completely eradicate triple-negative breast cancer tumors in both 2D and the spheroid cancer models. For studies related to hypoxia, it is necessary to perform expensive tests and most studies suggest using mouse models. In the present study, we confirmed that breast cancer spheroid provides hypoxia conditions in a controlled manner, and this hypoxia can be measured easily and at a low cost. In the same way, the function of nanoparticle or HAP drug can also be predicted.

 HAPs are very effective drugs against hypoxic tumors, although before using such strategies, it is necessary to ensure an optimized and suitable conditions for the effective action of the drug, or to use specific hypoxia markers such as pimonidazole. In this study, we used azolinker as a hypoxia-sensitive linker in the synthesis of nanoparticles, which is a toxic substance, and it is better to use natural and biocompatible crosslinkers in the synthesis of nanoparticles.

## Conclusion

 This study reports synthesis and formulation of novel NPs consisting of AU-PEI core and mPEG-AZO shell, as APAP NPs. The NP possesses two-stage hypoxia-activated potential: the first stage is the bioreduction of azo linker and the second stage is bioreduction of HAP drug. This first bioreduction results in the detachment of PEG shell, which exposes PEI core to efficiently transfect HAP into the hypoxic tumor region. Further, the localization of HAP in hypoxia favors its bioreduction to produce highly toxic oxygen radicals which effectively and locally kill resistant breast cancer cells. These nanoparticles with self-activation, biocompatibility, and targeted drug delivery potential have high therapeutic efficacy and significantly inhibit the growth of solid tumors in 2D and 3D cultured triple-negative breast cancer cells. This platform increases the efficacy while minimizing the toxicity of the TPZ, which is one of the common drugs in clinical phases II and III for advanced hypoxic tumors. It is expected that this nanoparticle will have broad therapeutic potential for a variety of advanced solid tumors with different degrees of hypoxia.

## Competing Interests

 None to declare.

## Data Availability Statement

 Not applicable.

## Ethical Approval

 This study is approved by NIMAD (IR.NIMAD.REC.1398.267).

## Supplementary Files


Supplementary file 1 contains Figure S1.
