# Supplementary material for: Tumor Microenvironment Responsive TPZ-Loaded Core-Shell Polymeric Nanoparticles for Selective Cancer Bioreductive Therapy
Source: Adv Pharm Bull. 2025 Jun 16;15(2):390–405. doi: 10.34172/apb.025.43945 (PMC12413963; doi:10.34172/apb.025.43945)
Supplement: Supplementary file 1 — contains Figure S1. [file apb-15-390-s001.pdf]

Adv Pharm Bull. 2025;15(2):390-S1
doi: 10.34172/apb.025.43945
https://apb.tbzmed.ac.ir

Supplementary file 1

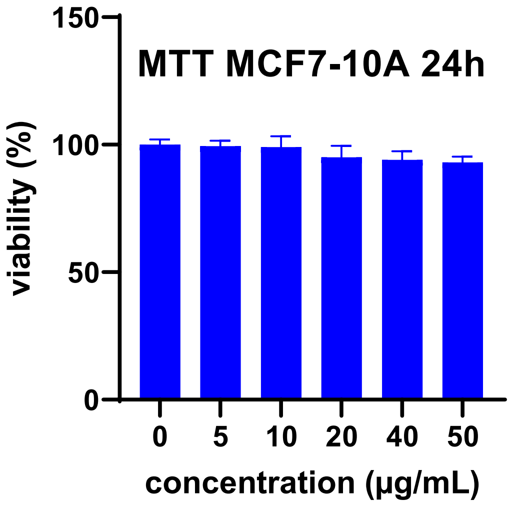

Figure S1: MTT assay of MCF7-10A normal breast cancer cells treated with APAP@TPZ for 24h.
